# Long-term physical activity leads to a significant increase in serum sRAGE levels: a sign of decreased AGE-mediated inflammation due to physical activity?

**DOI:** 10.1007/s00380-018-1125-5

**Published:** 2018-01-24

**Authors:** Michael Sponder, Ioana-Alexandra Campean, Michael Emich, Monika Fritzer-Szekeres, Brigitte Litschauer, Senta Graf, Daniel Dalos, Jeanette Strametz-Juranek

**Affiliations:** 1 Department of Cardiology, Medical University of Vienna, Währinger Gürtel 18-20, 1090 Vienna, Austria; 2 Austrian Federal Ministry of Defence and Sports, Austrian Armed Forces, Brünnerstraße 238, 1210 Vienna, Austria; 3 Department of Medical and Chemical Laboratory Analysis, Medical University of Vienna, Währinger Gürtel 18-20, 1090 Vienna, Austria; 4 Department of Pharmacology, Medical University of Vienna, Währinger Gürtel 18-20, 1090 Vienna, Austria

**Keywords:** sRAGE, RAGE, Advanced glycation end products, Physical activity, Inflammation

## Abstract

There is growing evidence that low levels of the circulating soluble receptor of advanced glycation end products (sRAGE) are a valuable predictor of cardiovascular disease (CVD). The aim of this prospective study was to investigate the influence of long-term physical activity on serum sRAGE levels. 109 subjects were recruited, and 98 completed the study. Participants were asked to perform exercise within the calculated training pulse for 8 months. The performance gain was measured/quantified by bicycle stress tests at the beginning and end of the observation period. sRAGE was measured at baseline and after 2/6/8 months by ELISA. Backwards, multiple linear regression analysis was performed to investigate the association of co-variables age, sex, BMI, and performance at baseline, HbA_1_c, and lipoprotein a with baseline sRAGE levels. We identified BMI and lipoprotein a as significant predictors for baseline sRAGE levels. Compared to subjects with a performance gain ≤ 4.9% subjects with a gain > 5% showed a significant increase in sRAGE levels up to 22%. sRAGE serum levels correlate negatively with lipoprotein a levels and BMI and long-term physical activity leads to a significant increase in serum sRAGE levels (9–22%), whereby the sRAGE increase is most pronounced in subjects with initially low-performance levels, suggesting that in particular, these subject profit the most from increased physical activity. The sport-mediated increase of sRAGE might be a sign of decreased AGE-mediated inflammation and highlight the protective effect of sports on CVD and other disease which are at least partly mediated by an increased inflammation status.

*Clinical trials registration* NCT02097199.

## Introduction

Advanced glycation end products (AGEs) have been associated with the prevalence of numerous disease, e.g., diabetes and its vascular complications [1], Alzheimer’s disease [2], and atherosclerosis [3]. The circulating soluble receptor of advanced glycation end products (sRAGE) is a biomarker reflecting RAGE (receptor for advanced glycation end products) activity. sRAGE levels were shown to be elevated after acute myocardial infarction (MI) [4], but lower levels of sRAGE are independently associated with incident coronary heart disease, diabetes mellitus, metabolic syndrome, heart failure, and death [5–7]. There is growing evidence that low sRAGE levels are a valuable predictor of cardiovascular disease. The investigation of the influence of long-term physical activity on biomarkers for calcification processes [8], inflammation [9] or the lipid profile [10, 11], inter alia by our group, has already delivered important information; however, the impact of long-term physical activity on sRAGE levels is mostly unknown and available data is conflicting [12, 13]. Therefore, it was the aim of the present prospective study to investigate the influence of 8 months of increased physical activity on serum sRAGE levels.

## Methods

In total, 109 subjects were recruited. The inclusion criteria were: age between 30 and 65 years and physical ability to perform endurance exercise. Exclusion criteria were: age < 30 or > 65 years, no ability to perform endurance exercise, current oncologic or infectious disease (anamnestic or increased inflammation parameters at baseline). 11 subjects did not complete the study for different reasons (accidents, loss of motivation, etc.). Finally, 98 subjects completed the study. The study population, therefore, consisted of 38 female and 60 male subjects aged 30–65 years with at least one classic cardiovascular risk factor: overweight (BMI > 25.0 kg/m^2^), hypertension (SBP > 140 ± DBP > 85 mmHg at rest/antihypertensive medication), hyper/dyslipidemia (anamnestic statin therapy), diabetes mellitus (HbA1c > 6.5 rel%/DM medication), current smoking, known CHD [anamnestic MI, PCI (percutaneous coronary intervention), CABG (coronary artery bypass graft), stroke], and positive family anamnesis for MI/CVD/stroke of mother and/or father. The anamnestic weekly alcohol intake was measured in units: 1 unit corresponds to 0.33 l beer, 0.125 l red/white wine, or 0.02 l spirits.

The study was carried out in adherence to the Declaration of Helsinki and its later amendments and the ethical standards in sports and exercise science research [14]. The protocol has been approved by the Ethical Commission of the Medical University of Vienna (EC-number: 1830/2013) and informed consent was obtained from all subjects before inclusion.

### Measurement of anthropometric data and bicycle stress test (ergometry)

After detailed anamnesis and physical examination including the measurement of height, weight, body water, body muscle mass, and body fat (with a diagnostic scale, Beurer BG 16, Beurer GmbH, Ulm, Germany), the subjects had to perform a bicycle stress test (ergometry) at the beginning of the study to define their performance level and to calculate their individual training pulse/target heart rate (using the Karvonen formula with an intensity level of 65–75% for moderate and 76–93% for vigorous intensity). The subjects were let to decide the kind of physical activity/sports; however, they were asked to perform at least 75 min/week of vigorous or 150 min/week of moderate intensity endurance training (or a mixture; strength training was allowed but not mandatory) within the calculated training pulse. A second ergometry was performed at the end of the study (after 8 months) to prove and also quantify exactly and objectively the change/gain in performance. Therefore, we relinquished the leading of a training protocol. The bicycle stress tests were always ECG-monitored and performed with the same system (Ergometer eBike comfort, GE Medical Systems, Freiburg, Germany) starting with 25 W and increasing every 2 min by 25 W (according to the protocol of the Austrian Society of Cardiology which is equal to the guidelines of the European Society of Cardiology). Blood pressure and heart rate were taken every 2 min. Subjects were told to cycle with 50–70 revolutions/min until exhaustion occurred. The target performance was calculated using body surface [calculated according to DuBois formula [15]: body surface (m^2^) = 0.007184 × height (cm)^0.725^ × weight (kg)^0.425^] [15], sex and age: for men: performance (W) = 6773 + 136,141 × body surface – 0.916 × body surface × age.

For women: performance (W) = 3933 + 86,641 × body surface – 0.346 × body surface × age.

An individual target performance of 100% represents the performance of an untrained collective. We estimate that a performance gain of at least 5% is necessary to manifest measurable and clinically relevant changes concerning the inflammation profile. Therefore, the study population was divided into 4 groups according to the baseline performance and to the performance gain over the observation period:

- Group 1 consists of participants with a performance ≤ 99% at baseline and a performance gain ≤ 4.9%.
- Group 2 of participants with a performance ≤ 99% at baseline and a performance gain > 5%.
- Group 3 of participants with a performance > 100% at baseline and a performance gain ≤ 4.9%.
- Group 4 of participants with a performance > 100% at baseline and a performance gain > 5%.

### Routine laboratory and sRAGE analysis

Blood samples were drawn in a not starving state. Blood samples for the determination of sRAGE (CycLex Co human sRAGE ELISA Kit, CY-8083 Nagano, Japan) were taken at baseline, after 2, 6, and 8 months and analysed by ELISA (enzyme-linked immunosorbent assay) according to the manufacturer’s instruction. The coefficient of variation (CV) was 6.9%. All blood samples were taken after 10 min of still lying from an arm vein with a tube/adapter system. Samples for the determination of routine laboratory parameters were analysed immediately after drawing. The analysis was performed according to the manufacturer’s instructions.

### Statistical analysis

Statistical analysis was accomplished using SPSS 20.0. Continuous and normally distributed data are described by mean ± standard deviation (SD). Non-normally distributed data are described by median/25th quartile/75th quartile. Single correlations involving only two normally distributed data were calculated using Pearson Correlation, single correlations involving two non-parametric data and/or ordinal data were calculated using Spearman’s rho analysis. To investigate the correlation of sRAGE levels with anthropometric data and laboratory parameters, we used Spearman’s rho analysis. Backwards, multiple linear regression analysis was performed to investigate the association of the co-variables age, sex, T2DM, smoking, BMI, performance gain, HbA_1_c and lipoprotein a with both baseline sRAGE levels and sRAGE levels after 8 months of physical activity.

As it was expected that not all of the subjects would reach an adequate performance gain during the observation period, we defined in the forefront a minimum threshold of 5% performance gain as significant and divided the study population into the 4 mentioned groups. To investigate trends over the observation period of 8 months, we used the Friedman test. All tests were performed in accordance with two-sided testing and *p* values ≤ 0.05 were considered significant.

## Results

### Risk factor profile, anthropometric, and laboratory data at baseline

Risk factor profile, anthropometric, and laboratory data for the four groups at baseline are shown in Table 1. Concerning the total population, the most prevalent cardiovascular risk factors were overweight/adipositas (65.3%), a positive family anamnesis (44.9%), hypertension (32.7%), and smoking (20.4%). Compared to the initially non-sportive groups 1 + 2, participants of the initially sportive groups, 3 + 4 had significantly higher body muscle mass and body water and lower body fat amount (35.6 ± 3.9 vs. 33.5 ± 3.9%, *p* = 0.014; 54.1 ± 6.1 vs. 49.9 ± 4.5%, *p* < 0.001; 27.5 ± 10.9 vs. 32.1 ± 6.2%, *p* = 0.018). There was no significant difference in BMI (28.3 ± 5.0 vs. 26.9 ± 3.4 kg/m^2^). The performance gain was − 0.4 ± 4.6% in group 1, 13.7 ± 6.7% in group 2, − 2.2 ± 5.3% in group 3, and 13.4 ± 4.9% in group 4.

**Table 1:** Description of population

	Initially non-sportive (*n* = 37)		Initially sportive (*n* = 61)			*p* value Group 1 + 2 vs. 3 + 4
	Group 1 (*n* = 14)	Group 2 (*n* = 23)	Group 3 (*n* = 27)		Group 4 (*n* = 34)	
Hypertension	*n* = 6/42.9%	*n* = 11/40.7%	*n* = 7/30.4%		*n* = 8/23.5%	
Dyslipidaemia	*n* = 4/28.6%	*n* = 7/25.9%	*n* = 8/34.8%		*n* = 10/29.4%	
Diabetes mellitus	*n* = 2/14.2%	*n* = 0/0%	*n* = 1/4.3%		*n* = 1/2.9%	
Overweight/adipose	*n* = 10/71.5%	*n* = 18/66.6%	*n* = 16/69.6%		*n* = 20/58.9%	
Ex-smoking	*n* = 5/35.7%	*n* = 11/40.7%	*n* = 14/60.9%		*n* = 12/35.3%	
Smoking	*n* = 6/42.9%	*n* = 7/25.9%	*n* = 3/13.0%		*n* = 4/11.8%	
Known CHD/stroke	*n* = 1/7.1%	*n* = 5/18.5%	*n* = 2/8.7%		*n* = 8/23.5%	
Family anamnesis	*n* = 7/50.0%	*n* = 13/48.1%	*n* = 11/47.8%		*n* = 13/38.2%	
Alcohol (units/week)	1.3 ± 1.7	2.9 ± 3.4	2.9 ± 3.7		3.4 ± 4.6	0.483
Age (years)	51 ± 5	48 ± 8	49 ± 7		49.7 ± 5.5	0.991
BMI (kg/m^2^)	28.2 ± 4.4	28.4 ± 5.3	27.1 ± 3.8		26.8 ± 3.1	0.298
Body water (%)	48 ± 3	51 ± 5	53 ± 6		55 ± 6	0.001**
Body fat (%)	34 ± 4	31 ± 7	28 ± 9		27 ± 12	0.002**
Body muscle (%)	32 ± 3	34 ± 4	34 ± 4		36 ± 4	0.013**
SBP (mmHg)	130 ± 11	134 ± 7	129 ± 16		132 ± 11	0.598
DBP (mmHg)	80 ± 9	78 ± 8	79 ± 9		77 ± 7	0.438
HR (bpm)	68 ± 9	69 ± 7	65 ± 9		65 ± 7	0.031**
Performance gain (%)	− 0.4 ± 4.6	13.7 ± 6.7	− 2.2 ± 5.3		13.4 ± 4.9	0.498
Erythrocytes (T/l)	4.7 ± 0.5	4.8 ± 0.4	4.6 ± 0.3		4.8 ± 0.4	0.921
Haemoglobin (g/dl)	13.8 ± 1.9	14.1 ± 1.3	13.7 ± 1.0		14.3 ± 1.1	0.997
Haematocrit (%)	40 ± 5	40 ± 3.2	40 ± 3		41 ± 3	0.683
Thrombocytes (G/l)	271 ± 67	241 ± 60	275 ± 68		242 ± 39	0.762
Leukocytes (G/l)	7.9 ± 2.0	6.4 ± 1.5	6.5 ± 1.8		6.1 ± 1.2	0.059
Na (mmol/l)	141 ± 2	141 ± 1.4	141 ± 2		142 ± 2	0.231
K (mmol/l)	4.1 ± 0.3	4.1 ± 0.3	4.3 ± 0.2		4.2 ± 0.3	0.028**
Cl (mmol/l)	101 ± 2	101 ± 2	101 ± 2.0		101 ± 2	0.542
Ca (mmol/l)	2.3 ± 0.1	2.3 ± 0.8	2.3 ± 0.8		2.3 ± 0.1	0.645
Phosphate (mmol/l)	1.1 ± 0.1	1.0 ± 0.1	1.1 ± 0.2		1.1 ± 0.2	0.043**
Mg (mmol/l)	0.83 ± 0.04	0.85 ± 0.06	0.8 ± 0.1		0.8 ± 0.1	0.563
Creatinine (mg/dl)	0.87 ± 0.16	0.8 ± 0.1	0.9 ± 0.2		0.9 ± 0.2	0.009**
BUN (mg/dl)	13.2 ± 3.6	20.0 ± 26.4	14.1 ± 3.9		14.1 ± 3.7	0.200
Uric acid (md/dl)	5.6 ± 2.1	5.1 ± 1.3	5.3 ± 1.5		5.3 ± 1.1	0.762
Lipasis (U/l)	35 ± 12	36 ± 14	42 ± 21		44 ± 18	0.005**
Cholinesterasis (kU/l)	8.1 ± 1.5	8.4 ± 1.7	7.8 ± 1.7		8.4 ± 1.7	0.721
Alkalic phosphate (U/l)	64 ± 19	71 ± 65	57 ± 16		59 ± 14	0.320
ASAT (U/l)	28 ± 9	23 ± 8	27 ± 7		24 ± 5	0.277
ALAT (U/l)	29 ± 15	28 ± 15	26 ± 9		25 ± 9	0.728
Gamma-GT (U/l)	25 ± 15	39 ± 76	21 ± 12		27 ± 19	0.709
LDH (U/l)	179 ± 27	173 ± 27	175 ± 28		170 ± 21	0.656

Cardiovascular risk factor profile, anthropometric data and lab-analysis at baseline of the four groups

*CHD* chronic heart disease, *BMI* body mass index, *SBP* systolic blood pressure, *DBP* diastolic blood pressure, *HR* heart rate, *Na* natrium, *K* potassium, *Cl* chloride, *Ca* calcium, *Mg* magnesium, *BUN* blood urea nitrogen, *ASAT* aspartate aminotransferasis, *ALAT* alanine aminotransferasis, *GT* glutamyl transferasis, *LDH* lactate dehydrogenasis

The *p* values represent the significance of the differences between baseline values of the initially non-sportive groups (Group 1 + 2) vs. the initially sportive groups (Group 3 + 4). For better recognisability the significant differences are marked (**)

Concerning the total population, at baseline, sRAGE levels correlated positively with chloride (*p* = 0.039) and anorganic phosphate (*p* = 0.049) and negatively with BMI (*p* = 0.006), erythrocyte count (*p* = 0.018), haemoglobin (*p* < 0.001), haematocrit (*p* = 0.001), ALAT (*p* = 0.019), apolipoprotein B (*p* = 0.037), and lipoprotein a (*p* = 0.006). Concerning other anamnestic, anthropometric, and laboratory parameter, there was no correlation; in particular, sRAGE levels did not correlate with smoked pack years (*p* = 0.727), weekly alcohol intake (*p* = 0.760), age (*p* = 0.323), hsCRP (*p* = 0.540), interleukin-6 (*p* = 0.145), HbA1_c_ (*p* = 0.284), and C-peptide (*p* = 0.079). Backwards, multiple linear regression analysis was performed to investigate the association of the co-variables age, sex, BMI, performance gain, HbA_1_c, and lipoprotein a with baseline sRAGE levels. We identified BMI and lipoprotein a as (borderline) significant predictors for baseline sRAGE levels and also sRAGE levels after 8 months of physical activity, as shown in Table 2 (baseline: *F* = 6.08; *p* < 0.005; $r_{\text{adj}}^{2}$ = 0.10; after 8 months of physical activity: *F* = 4.63; *p* < 0,012; $r_{\text{adj}}^{2}$ = 0.07).

**Table 2:** Backwards multiple linear regression analysis

	Regression coefficient *B*^b^	Standard error	*β*	*T*	Significance
At baseline					
Constant	578.547				
Lipoprotein a	− 0.494	0.249	− 0.206	− 1.986	0.050
BMI	− 6.677	3.386	− 0.205	− 1.972	0.052
*F* = 6.08; *p* < 0.005; $r_{\text{adj}}^{2}$ = 0.10					
After 8 months					
Constant	630.207				
Lipoprotein a	− 0.419	0.246	− 0.174	− 1.705	0.091
BMI	− 7.407	3.772	− 0.201	− 1.964	0.053
*F* = 4.63; *p* < 0.012; $r_{\text{adj}}^{2}$ = 0.07					

Backwards multiple linear regression analysis; age, sex, smoking, T2DM, BMI, performance gain, HbA_1_c and lipoprotein a were inserted as co-variables but only BMI and lipoprotein a were significant predictors of baseline sRAGE levels and sRAGE levels after 8 months of physical activity

### Influence of physical activity on sRAGE levels

There was no significant difference concerning baseline sRAGE and hsCRP levels for group 1 + 2 vs. group 3 + 4 (*p* = 0.172 and 0.077), but there was a significant difference for IL-6 (*p* = 0.020). The sRAGE levels at the different points of measurement of the four groups are shown in Table 3. The initially sportive participants (group 3 + 4) showed higher sRAGE levels at baseline compared to the initially non-sportive groups (group 1 + 2). There was no significant change in sRAGE levels in group 1 (*p* = 0.392; − 8.3%) and group 3 (*p* = 0.184; 5.8%) compared to significant changes in group 2 (*p* = 0.015; 22.4%) and group 4 (*p* < 0.001; 9.3%) calculated by Friedman test. We also stated a decrease of the inflammation parameters hsCRP and IL-6 in group 2 and group 4 but without statistical significance.

**Table 3:** Progression of sRAGE, hsCRP, IL-6, HbA1_c,_ and C-peptide

	Initially non-sportive (*n* = 37)		Initially sportive (*n* = 61)	
	Group 1 (*n* = 14)	Group 2 (*n* = 23)	Group 3 (*n* = 27)	Group 4 (*n* = 34)
sRAGE baseline	339/258/380	330/280/367	346/275/480	366/288/481
sRAGE 2 months	330/252/450	375/285/437	429/310/465	409/292/505
sRAGE 6 months	366/246/437	392/321/488	424/316/543	401/296/519
sRAGE 8 months	311/228/448	404/311/442	366/316/504	400/322/489
Change (%)	− 8.3	22.4	5.8	9.3
*p* value	0.392	0.015	0.184	< 0.001
hsCRP baseline	0.09/0.05/0.22	0.14/0.08/0.31	0.11/0.05/0.23	0.09/0.06/0.12
hsCRP 2 months	0.15/0.05/0.24	0.13/0.07/0.26	0.09/0.07/0.27	0.08/0.04/0.17
hsCRP 6 months	0.07/0.05/0.21	0.15/0.06/0.26	0.14/0.05/0.36	0.09/0.05/0.17
hsCRP 8 months	0.18/0.08/0.27	0.11/0.05/0.27	0.12/0.06/0.25	0.07/0.04/0.11
*p* value	0.532	0.076	0.567	0.113
IL-6 baseline	3.2/1.9/3.6	2.6/1.8/3.2	1.9/1.5/3.0	2.1/1.5/2.9
IL-6 2 months	2.4/1.7/2.8	2.4/0.0/3.4	1.7/1.5/2.3	1.9/1.5/2.6
IL-6 6 months	2.1/1.5/2.9	2.3/1.6/3.0	2.4/0.8/3.3	1.8/1.5/2.8
IL-6 8 months	2.2/0.8/3.0	2.1/0.0/3.4	1.7/0.8/2.4	1.7/1.1/2.6
*p* value	0.169	0.138	0.197	0.397
HbA1_c_ baseline	5.8 ± 1.0	5.2 ± 0.3	5.4 ± 0.8	5.2 ± 0.3
HbA1_c_ 2 months	5.8 ± 0.9	5.2 ± 2.3	5.3 ± 0.7	5.2 ± 0.3
HbA1_c_ 6 months	5.8 ± 1.0	5.2 ± 0.3	5.5 ± 0.7	5.2 ± 0.3
HbA1_c_ 8 months	5.9 ± 0.9	5.2 ± 0.3	5.4 ± 0.5	5.3 ± 0.3
*p* value	0.260	0.134	< 0.001	0.259
C-peptide baseline	4.6 ± 4.3	3.6 ± 2.7	3.1 ± 2.0	3.3 ± 2.4
C-peptide 2 months	4.1 ± 3.7	3.8 ± 2.2	3.4 ± 1.8	3.9 ± 2.6
C-peptide 6 months	3.8 ± 2.7	4.4 ± 2.8	3.2 ± 2.1	3.6 ± 2.2
C-peptide 8 months	4.5 ± 4.6	3.6 ± 2.0	3.1 ± 1.5	3.6 ± 2.1
*p* value	0.856	0.162	0.910	0.822

Progression of sRAGE, hsCRP, Il-6, HbA1_c_ and C-peptide in the four groups at the four points of measurement (baseline, after 2, 6 and 8 months). Data is given as median/25th/75th percentile for non-parametric values and as mean ± std dev for normally distributed values. The significances of the progressions (*p* value) have been calculated using Friedman test. The change in sRAGE levels (baseline vs. value after 8 months) is also described in percent

*sRAGE* soluble receptor of advanced glycation end products, *hsCRP* high-sensitivity C-reactive protein, *IL-6* interleukin-6

## Discussion

We could show a negative correlation of lipoprotein and sRAGE levels at baseline and a significant physical activity-mediated increase in serum sRAGE levels and the sRAGE increase was most pronounced in subjects with initially low-performance levels. AGEs occur when reducing sugar reacts with amino acids in proteins or other macromolecules. AGEs are mainly generated via the Maillard reaction; however, they can also be formed by oxidation of glucose or peroxidation of lipids [16]. High AGEs levels have been associated with aging [17] and disease which are related to aging such as diabetes and its vascular complications [1], Alzheimer’s disease [2], and atherosclerosis [3]. The interaction of AGEs with the receptor for advanced glycation end products (RAGE), a transmembrane receptor of the immunoglobulin superfamily composed of three domains which is expressed by, e.g., endothelial cells, lymphocytes, monocytes, and vascular smooth muscle cells, triggers the activation of mitogen-activated protein kinases (MAPKs) and phosphatidylinositol-3 kinase (PI3-K) leading to the activation of nuclear factor kappa B (NF-κB). Finally, NF-κB activation leads to a release of inflammatory cytokines like tumour necrosis factor α (TNFα), interleukin 6 (Il-6). Furthermore, a positive feedback mechanism between NF-κB and RAGE has been described [18]. Consequently, the interactions of AGEs with RAGE result in oxidative stress and the production of matrix metalloproteinases (MMPs), which cleave RAGEs and produce sRAGE [19].

sRAGE, the circulating soluble receptor of advanced glycation end products, is used as biomarker reflecting RAGE activity. sRAGE competes with RAGE by acting as a decoy receptor and may, therefore, prevent inflammatory processes [19]. sRAGE was shown to be elevated after myocardial infarction [4] and increased sRAGE levels were associated with poor in-hospital prognosis [20]. The major source of sRAGE in acute coronary syndrome (ACS) is not clear; however, Jensen et al. [21] speculate that it originates from the cardiomyocytes or the vascular cells in the damaged myocardium. Consequently, it seems that sRAGE is upregulated in current inflammatory processes (e.g., after myocardial infarction) to counteract inflammation-based damage. On the other hand, studies by Selvin et al. [5], Momma et al. [6], and Lazo et al. [7] could show that lower levels of sRAGE are independently associated with incident coronary heart disease, diabetes mellitus, metabolic syndrome, heart failure, and death. It seems that sRAGE levels are upregulated in acute settings such as myocardial infarction, but also in chronic disease, high sRAGE levels are beneficial. We could show a physical activity-mediated increase of sRAGE levels, whereby in particular, subjects with an initially low level of physical performance benefitted by an increase of up to 22%. Furthermore, data from the ARIC study [22] delivered results that showed an inverse relationship of sRAGE with current inflammation markers (hsCRP, fibrinogen). However, we neither observed a positive nor a negative correlation of sRAGE with hsCRP or Il-6 at baseline.

Glucose plays an important role in sRAGE metabolism. AGEs accumulate in human tissue proteins as a result of chronic hyperglycemia, and since sRAGE levels have been negatively correlated with serum glucose [23, 24], we also analysed the HbA1_c_ and C-peptide progression over the observation period. However, although groups 2 and 4 showed a significant increase in sRAGE levels, there was no change in HbA1_c_ and C-peptide observable. However, we could show a negative correlation of lipoprotein a, a lipoprotein which is involved in the pathogeneses of vascular diseases, with sRAGE.

Studies in human dealing with the influence of long-term physical activity on AGE [25, 26] and sRAGE levels are rare and conflicting: Kotani et al. [12] measured serum sRAGE levels in 15 female and 15 male healthy, non-smoking Japanese volunteers (mean age 65 years) in the pre- and post-phase of a 6-month interventional program and stated a significant reduction of circulating sRAGE levels. However, with 30 individuals, the sample size was very small and the training program only included monthly explanatory and motivational sessions to increase physical activity such as daily walking and to instruct on the appropriate methods. The participants were educated on how to measure their heart rates during a walking exercise in their radial arterial pulses, and recommended to perform the walking at 60% of the maximal heart rate for at least ≥ 30 min. Choi et al. [13] investigated the influence of physical activity on sRAGE in 75 female non-smoking patients (mean age 55 years) suffering diabetes mellitus. They performed a bicycle stress test at the beginning of the study and the 12-week-exercise program (the subjects were assigned to walk for 60 min per session, five times per week at moderate exercise capacity) was monitored by a multirecord accelerometer. This study group finally observed a significant increase of circulating sRAGE levels.

It is known for a long time that physical activity is one of the most potent therapeutic intervention to prevent CVD or at least to retard its progression. The activation of the AGE–RAGE axis results in the generation of inflammatory cytokines and oxidative stress. However, low sRAGE levels have been associated with coronary heart disease, diabetes mellitus, metabolic syndrome, heart failure, and death, because sRAGE acts as decoy for RAGE ligands and thus has cytoprotective effects. The increase of serum sRAGE levels in sporting individuals might be a sign of a physical activity-derived decrease in AGE/RAGE-mediated inflammation and might partly be involved in the protective effect of sports on the vasculature. However, although the chosen inflammation parameters (IL-6, hsCRP) decreased in the both groups with proven performance gain (and increasing sRAGE levels), the decrease of the inflammation parameters was not significant, probably due to the low inflammation level at baseline.

## Conclusion

sRAGE serum levels correlate negatively with lipoprotein a levels and BMI and long-term physical activity leads to a significant increase in serum sRAGE levels (9–22%), whereby the sRAGE-increase is most pronounced in subjects with initially low-performance levels, suggesting that in particular, these subjects profit the most from increased physical activity. The sports-mediated increase of sRAGE might be a sign of decreased AGE-mediated inflammation and highlight the protective effect of sports on CVD and other disease which are at least partly mediated by an increased inflammation status.

## Limitations

The present study has several limitations: First, although 98 participants completed the study the number of subjects is too low to show sex-specific differences. Second, several other not controlled influences and circumstances (besides physical activity) might have led to an increase in sRAGE levels.

## Abbreviations

AGE: Advanced glycation end products
ALAT: Alanine aminotransferase
ASAT: Aspartate aminotransferase
BMI: Body mass index
CABG: Coronary artery bypass graft
CHD: Chronic heart disease
CV: Coefficient of variation
CVD: Cardiovascular disease
DBP: Diastolic blood pressure
ELISA: Enzyme-linked immunosorbent assay
GT: Glutamyl transferase
hsCRP: High-sensitivity C-reactive protein
IL: Interleukin
LDH: Lactate dehydrogenase
MAPKs: Mitogen-activated protein kinases
MI: Myocardial infarction
MMP: Matrix metalloproteinases
NF-κB: Nuclear factor kappa B
PCI: Percutaneous coronary intervention
PI3-K: Phosphatidylinositol-3 kinase
RAGE: Receptor for advanced glycation end products
SBP: Systolic blood pressure
SD: Standard deviation
sRAGE: Soluble receptor of advanced glycation end products
